# Supplementary material for: Three-dimensionally preserved soft tissues and calcareous hexactins in a Silurian sponge: implications for early sponge evolution
Source: R Soc Open Sci. 2019 Jul 31;6(7):190911. doi: 10.1098/rsos.190911 (PMC6689616; doi:10.1098/rsos.190911)
Supplement: Table S1. Measurements of choanocyte chamber diameter. [file rsos190911supp1.docx]

**Supplementary Information**

Nadhira A, Sutton MD, Botting JP, Muir LA, Gueriau P, King A, Briggs DEG, Siveter David J, Siveter Derek J. Three-dimensionally preserved soft-tissues and calcareous hexactins in a Silurian sponge: implications for early sponge evolution. *Royal Society Open Science.*

**Table S1.** Measurements of choanocyte chamber diameter.

| Chamber | Width/px | Height/px | Width/µm | Height/µm |
| --- | --- | --- | --- | --- |
| 1 | 14 | 9 | 133.3 | 85.7 |
| 2 | 11 | 8 | 104.8 | 76.2 |
| 3 | 14 | 12 | 133.3 | 114.3 |
| 4 | 9 | 9 | 85.7 | 85.7 |
| 5 | 11 | 9 | 104.8 | 85.7 |
| 6 | 9 | 8 | 85.7 | 76.2 |
| 7 | 10 | 8 | 95.2 | 76.2 |
| 8 | 12 | 11 | 114.3 | 104.8 |
| 9 | 14 | 10 | 133.3 | 95.2 |
| 10 | 11 | 8 | 104.8 | 76.2 |
| 11 | 12 | 10 | 114.3 | 95.2 |
| 12 | 7 | 8 | 66.7 | 76.2 |
| 13 | 13 | 9 | 123.8 | 85.7 |
| 14 | 9 | 9 | 85.7 | 85.7 |
| 15 | 9 | 7 | 85.7 | 66.7 |
| 16 | 8 | 6 | 76.2 | 57.1 |
| 17 | 9 | 9 | 85.7 | 85.7 |
| 18 | 9 | 6 | 85.7 | 57.1 |
| 19 | 12 | 11 | 114.3 | 104.8 |
| 20 | 8 | 7 | 76.2 | 66.7 |
| 21 | 6 | 8 | 57.1 | 76.2 |
| 22 | 10 | 9 | 95.2 | 85.7 |
| 23 | 8 | 8 | 76.2 | 76.2 |
| 24 | 8 | 7 | 76.2 | 66.7 |
| 25 | 9 | 7 | 85.7 | 66.7 |
| 26 | 9 | 8 | 85.7 | 76.2 |
| 27 | 7 | 7 | 66.7 | 66.7 |
| 28 | 10 | 9 | 95.2 | 85.7 |
| 29 | 11 | 8 | 104.8 | 76.2 |
| 30 | 11 | 9 | 104.8 | 85.7 |
| 31 | 9 | 8 | 85.7 | 76.2 |
| 32 | 7 | 7 | 66.7 | 66.7 |
| 33 | 8 | 7 | 76.2 | 66.7 |
| 34 | 10 | 8 | 95.2 | 76.2 |
| 35 | 8 | 8 | 76.2 | 76.2 |
| 36 | 7 | 7 | 66.7 | 66.7 |
| 37 | 11 | 8 | 104.8 | 76.2 |
| 38 | 7 | 7 | 66.7 | 66.7 |
| 39 | 11 | 9 | 104.8 | 85.7 |
| 40 | 9 | 8 | 85.7 | 76.2 |
| 41 | 9 | 8 | 85.7 | 76.2 |
| 42 | 9 | 9 | 85.7 | 85.7 |
| 43 | 7 | 9 | 66.7 | 85.7 |
| 44 | 12 | 8 | 114.3 | 76.2 |
| 45 | 8 | 8 | 76.2 | 76.2 |
| 46 | 7 | 8 | 66.7 | 76.2 |
| 47 | 8 | 8 | 76.2 | 76.2 |
| 48 | 8 | 9 | 76.2 | 85.7 |
| 49 | 9 | 10 | 85.7 | 95.2 |
| 50 | 12 | 10 | 114.3 | 95.2 |
| 51 | 9 | 8 | 85.7 | 76.2 |
| 52 | 7 | 8 | 66.7 | 76.2 |
| 53 | 7 | 7 | 66.7 | 66.7 |
| 54 | 10 | 11 | 95.2 | 104.8 |
| 55 | 9 | 11 | 85.7 | 104.8 |
| 56 | 9 | 9 | 85.7 | 85.7 |
| 57 | 8 | 7 | 76.2 | 66.7 |
| 58 | 7 | 7 | 66.7 | 66.7 |
| 59 | 8 | 8 | 76.2 | 76.2 |
| 60 | 6 | 5 | 57.1 | 47.6 |
| 61 | 5 | 7 | 47.6 | 66.7 |
| 62 | 7 | 6 | 66.7 | 57.1 |
| 63 | 6 | 7 | 57.1 | 66.7 |
| 64 | 11 | 11 | 104.8 | 104.8 |
| 65 | 6 | 5 | 57.1 | 47.6 |
| 66 | 7 | 7 | 66.7 | 66.7 |
| 67 | 9 | 12 | 85.7 | 114.3 |
| 68 | 10 | 9 | 95.2 | 85.7 |
| 69 | 7 | 6 | 66.7 | 57.1 |
| 70 | 6 | 7 | 57.1 | 66.7 |
| 71 | 8 | 9 | 76.2 | 85.7 |

**Table S1**. Measurements of 71 different sediment-filled soft-tissue chambers (interpreted as choanocyte chambers) from physical-optical tomograms of OUMNH C.36032. Heights and widths measured in pixels (px) and converted to microns (µm); tomogram resolution was 105 pixels/mm. Mean diameter (i.e. mean of all heights and widths) 82.0 µm, standard deviation 17.5 µm, range 48-133 µm.
